# Twenty-Four Hour Glucose Profiles and Glycemic Variability during Intermittent Religious Dry Fasting and Time-Restricted Eating in Subjects without Diabetes: A Preliminary Study

**DOI:** 10.3390/nu16162663

**Published:** 2024-08-12

**Authors:** Beeke Peters, Christina Laetitia Pappe, Daniela A. Koppold, Katharina Schipp, Bert Arnrich, Andreas Michalsen, Henrik Dommisch, Nico Steckhan, Olga Pivovarova-Ramich

**Affiliations:** 1 Department of Molecular Metabolism and Precision Nutrition, German Institute of Human Nutrition Potsdam-Rehbruecke, 14558 Nuthetal, Germany; 2 German Center for Diabetes Research (DZD), 85764 Neuherberg, Germany; 3 Charité-Universitätsmedizin Berlin, Corporate Member of Freie Universität Berlin and Humboldt-Universität zu Berlin, Department of Periodontology, Oral Medicine and Oral Surgery, 10117 Berlin, Germany; 4 Charité-Universitätsmedizin Berlin, Corporate Member of Freie Universität Berlin and Humboldt-Universität zu Berlin, Institute of Social Medicine, Epidemiology and Health Economics, 10117 Berlin, Germany; 5 Department of Internal and Integrative Medicine, Immanuel Hospital Berlin, 14109 Berlin, Germany; 6 Institute of Nutritional Medicine, University of Lübeck, 23538 Lübeck, Germany; 7 Digital Health-Connected Healthcare, Hasso Plattner Institute, University of Potsdam, 14469 Potsdam, Germany; 8 Evidence-Based Digital Diabetology, Medical Faculty Carl Gustav Carus, Department of Medicine III, Prevention and Care of Type 2 Diabetes, Technical University of Dresden, 01307 Dresden, Germany; 9 Charité-Universitätsmedizin Berlin, Corporate Member of Freie Universität Berlin and Humboldt-Universität zu Berlin, Department of Endocrinology and Metabolism, 10117 Berlin, Germany

**Keywords:** continuous glucose monitoring, glucose metabolism, glycemic variability, religious fasting, intermittent fasting, time-restricted eating

## Abstract

Intermittent religious fasting increases the risk of hypo- and hyperglycemia in individuals with diabetes, but its impact on those without diabetes has been poorly investigated. The aim of this preliminary study was to examine the effects of religious Bahá’í fasting (BF) on glycemic control and variability and compare these effects with time-restricted eating (TRE). In a three-arm randomized controlled trial, 16 subjects without diabetes were assigned to a BF, TRE, or control group. Continuous glucose monitoring and food intake documentation were conducted before and during the 19 days of the intervention, and the 24 h mean glucose and glycemic variability indices were assessed. The BF and TRE groups, but not the control group, markedly reduced the daily eating window while maintaining macronutrient composition. Only the BF group decreased caloric intake (−677.8 ± 357.6 kcal, *p* = 0.013), body weight (−1.92 ± 0.95 kg, *p* = 0.011), and BMI (−0.65 ± 0.28 kg, *p* = 0.006). Higher maximum glucose values were observed during BF in the within-group (+1.41 ± 1.04, *p* = 0.039) and between-group comparisons (BF vs. control: *p* = 0.010; TRE vs. BF: *p* = 0.022). However, there were no alterations of the 24 h mean glucose, intra- and inter-day glycemic variability indices in any group. The proportions of time above and below the range (70–180 mg/dL) remained unchanged. BF and TRE do not exhibit negative effects on glycemic control and variability in subjects without diabetes.

## 1. Introduction

Intermittent fasting (IF), characterized by alternating periods of voluntary fasting and eating, has gained popularity in recent years [1]. Preclinical and clinical studies on IF have demonstrated various health benefits, including its effectiveness for weight loss [1], although the findings are heterogenous and dependent on the form of IF and the animal model or study cohort. Notably, IF can also induce negative health effects. In particular, religious Ramadan fasting (RF) showed an amelioration of glycemic control [2,3].

Bahá’í fasting (BF) is a form of religious intermittent dry fasting, similar to RF, characterized by restrictions on food and fluid intake from sunrise to sunset [4]. Followers of the Bahá’í religion observe fasting as a significant spiritual duty, fasting for 19 consecutive days every year in March [5], whereas RF lasts for 29 or 30 days. In contrast to RF, where the fasting duration varies based on the lunar calendar and region [6], BF is performed when days and nights are approximately equal (at the equinox) [5]. This makes BF a more stable model for exploring the effects of intermittent dry fasting compared to RF.

Religious intermittent dry fasting involves significant changes in diurnal eating and sleeping patterns, water balance, and hormonal patterns [7]. In subjects with diabetes, particularly those receiving insulin therapy, these alterations may increase the risk of the deterioration of glycemic control. Because only a few studies have investigated the BF impact on metabolism [5,8], the effects of religious intermittent dry fasting on the glycemic control described below are based on RF studies. Published studies have reported that individuals with type 1 and type 2 diabetes have an increased risk of hypo- and hyperglycemic events during religious intermittent dry fasting in the form of RF [2,3,9,10]. Severe hypoglycemia was more frequent in the subjects who were treated with insulin and/or sulfonylureas, changed their dose of oral antidiabetic drugs or insulin, or modified their level of physical activity [2,3,10]. Despite these risks, some studies have reported improvements in HbA1c, fasting and postprandial glucose, body weight, and body fat after RF [11,12].

The development and increasing availability of continuous glucose monitoring (CGM) for 24 h glucose patterns offer new opportunities for such investigations. In particular, CGM allows an assessment of glycemic variability (GV), which includes the amplitude, frequency, and duration of glycemic fluctuations around the mean blood glucose. GV is recognized as an emerging target for blood glucose control [13] and an independent risk factor for micro- and macrovascular complications in subjects with and without diabetes [14]. Recent studies using CGM (or flash glucose monitoring, FGM) during RF have confirmed an increased time in hyperglycemia and glucose variability, especially in subjects with type 1 diabetes receiving insulin therapy [15,16,17]. In contrast, subjects with type 2 diabetes with or without insulin treatment showed only a transient increase in glucose variability and no changes in glycemic control measures during RF [18,19]. Nevertheless, there is still a lack of sufficiently controlled CGM-based data on the impact of RF on glycemic control.

Time-restricted eating (TRE) is another form of IF that has gained popularity in recent years. It involves prolonged daily fasting and a reduction in the daily eating window usually to under 10 h/day, with variations in the timing and duration of it (e.g., early, midday, or late TRE) [20]. In contrast to RF, most TRE trials reported beneficial effects on metabolic outcomes, even though the data are strongly heterogeneous [21]. A number of TRE studies showed improvements in glucose metabolism, such as reductions in fasting, postprandial, and nocturnal glucose levels; lowered mean 24 h glucose levels; reduced glycemic excursions; improvements in HbA1c; reduced fasting insulin levels; insulin resistance; and improved insulin sensitivity [21]. However, whether TRE is comparable to the intermittent religious dry fasting regimes regarding their effects on glucose patterns remains unclear.

Religious intermittent dry fasting is obligatory for healthy adult Muslims (in the form of RF) or followers of the Bahá’í religion (in the form of BF) and the desire to participate is intense, even in groups at risk, e.g., those with diabetes [10]. Managing diabetes during religious fasting is challenging, as taking countermeasures in the case of hypoglycemia would lead to breaking the fast. Therefore, the question of whether or to what extent glucose metabolism could be out of control, especially with regard to glucose variability, in individuals without diabetes [22] has high clinical relevance and needs to be carefully investigated. Therefore, the main aim of this preliminary study was to examine how religious BF affects glycemic control and variability in adult individuals without diabetes and whether the effects are comparable to TRE.

## 2. Materials and Methods

### 2.1. Study Design

**Figure 1:**
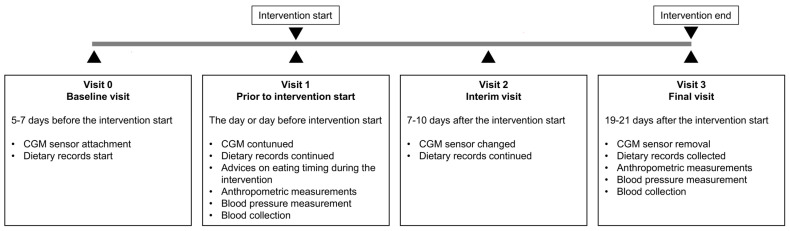
Study design. Intervention start indicates the beginning of the intermittent fasting in the BF and TRE groups, whereas the control group was instructed not to alter its habitual food and eating timing.

This three-arm randomized, controlled trial investigated the effects of two different fasting regimes, the religious dry BF and TRE, compared to a control group. After a 5–7 day baseline phase, the intervention groups followed a corresponding fasting regimen for 19 days, whereas the control group did not receive any nutritional recommendations and was advised not to change its habitual food and eating timing (Figure 1). Before and at the end of the intervention phase, the anthropometric parameters and blood pressure were assessed and a blood sample was collected (visit 1 and visit 3). HbA1c was measured at visit 1 to determine the glycemic status of the study subjects. CGM and food intake documentation (dietary records) were conducted simultaneously during the baseline phase and during the entire intervention (Figure 1).

The trial was designed as a dental study with the bleeding on probing index as the primary periodontal outcome parameter (Supplemental Figure S1). Here, we focus on the secondary outcomes related to glycemic control in a sub-cohort of 16 participants who underwent CGM assessment over the whole study duration. This study was approved by the ethics committee of the Charité—Universitätsmedizin Berlin on 6 May 2021, registered on the German Clinical Trials register (DRKS, assessed on drks.de) under the identifier number DRKS00026701 on 15 October 2021 and conducted between November 2021 and May 2022. The recruitment of the participants was performed in the Department for Periodontology, Oral Medicine, and Oral Surgery at Charité—Universitätsmedizin Berlin, Germany. Written informed consent was obtained from all the study participants prior to this study.

### 2.2. Study Subjects and Eligibility Criteria

The study participants were males and females without periodontal disease aged between 18 and 69. For the BF group, the participants had to be a member of the Bahá’í community who intended to fast. The subjects were randomly assigned to either the TRE or control groups, stratified by gender, with random block sizes of 2 or 4 (blockrand backage in R Studio version, 2022.12.01), whereas the BF group participants were not randomized. The exclusion criteria were diabetes; pregnancy and breastfeeding; severe internal diseases; eating disorders and severe psychiatric illness; smoking (defined as >5 cigarettes daily); caries; periodontitis; ongoing orthodontic therapy; other pathological oral conditions; and medication with antibiotics (in the last three months prior to the study start) or anti-inflammatory drugs (during the fasting period). Moreover, the participants were excluded when participating in another intervention study or planning to interrupt their (religious) fasting.

### 2.3. Fasting Interventions, Dietary Records, and Assessment of Timely Compliance

During the intervention phase, the participants of the BF group only consumed food and drink before sunrise and after sunset, without any fluid intake during the day (dry fasting). In the TRE group, the participants were advised to consume their habitual food and caloric drinks in a restricted eating window of 8 h a day, followed by a 16 h fasting window when only non-caloric drinks (e.g., water and herbal tea) were allowed. The participants were free to select their own mealtimes as long as they adhered to the 8 h eating and 16 h fasting windows.

The participants were asked to document all consumed foods and drinks and the eating times during the baseline and intervention phases starting from visit 0 (Figure 1). They were instructed to weigh their food whenever possible, write down brand names, and use standard household measures (e.g., cups, glasses, tablespoon, teaspoon, etc.) when they go out for dinner. Dietary records during the baseline and intervention phases were analyzed for the daily energy and macronutrient intake, as well as for the eating timing, using the FDDB database (Fddb Internetportale GmbH, https://fddb.info/ (accessed on 4 November 2022)) as described [23,24]. Furthermore, the dietary glycemic index (GI) was calculated for each day of the baseline and intervention for all the participants as described [25,26].

For the assessment of dietary compliance, in the TRE group, the beginning of the first and the end of the last caloric intakes were determined based on the dietary records, and the average daily eating and fasting window durations were calculated for each participant for the baseline and intervention phases. In the BF group, the subjects usually ate two times per day—before sunrise (=after waking up) and after sunset (=before bedtime). To calculate the daily eating and fasting windows, the time between the first and last caloric intake in the morning and the first and last caloric intake in the evening were summed. In the BF group, the adherence to the sunrise and sunset times was additionally assessed using the sunrise–sunset calendar specific for every fasting day. Adherence to 8 h eating windows during the TRE intervention and to specific sunrise and sunset times in the BF intervention were considered as compliant with a 30 min tolerance window. Compliant days were calculated as a percentage of the number of all intervention days.

### 2.4. Anthropometric Measurements

Anthropometric measurements, e.g., body weight and body composition, were performed using a scale and the body analyzer BF 508 (Omron, Mannheim, Germany). Waist and hip circumferences were assessed with a measurement tape.

### 2.5. Continuous Glucose Monitoring (CGM) and HbA1c Assessment

Twenty-four hour continuous glucose monitoring was performed with the CGM system of FreeStyle Libre Pro IQ (Abbott, Wiesbaden, Germany). The glucose sensor was placed on the upper arm and measured the interstitial blood glucose in 15 min sampling intervals. The sensor was blinded, and, therefore, participants were not able to check their blood glucose values. The first CGM recording period started 5–7 days before the intervention start (visit 0) and continued for 14 consecutive days. On the 7–10th days of the intervention (visit 2), the new sensor was fixed, and the second CGM recording period continued till the final visit at the end of the intervention (visit 3) (Figure 1).

The sensor data were read by a study assistant and analyzed by the Excel tool EasyGV Version 9.0.R2 [27], quantifying an average 24 h mean sensor glucose level (MSG) and the relevant indices describing glycemic variability during the baseline and intervention phases as described [24]. To analyze the intra-day glycemic variability, the standard deviation (SD) was calculated for each mean glucose value as described [27]. The mean amplitude of glucose excursions (MAGE) was determined by SD values higher than 1 [27]. The inter-day glycemic variability was described using the continuous overlapping net glycemic action (CONGA) as the index for the glucose difference at various set intervals, with its length being set at 60 min prior to the analysis. The mean of absolute glucose change (MAG) was calculated as a sum of the consecutive glucose level differences divided by the total time (hours) [28]. The mean of daily differences (MODD), which is an index describing the inter-day glycemic variability, was calculated with the averaged glucose values occurring at the same time on different days. Moreover, a classification of the glucose values and their conversion to risk scores, e.g., a low blood glucose index (LBGI < 0) and a high blood glucose index (HBGI > 0), was made. The coefficient of variation in percentage (CV%) was calculated using the formula SD/MSG × 100 as described previously [29]. The time below range (TBR, percentage of glucose values < 3.9 mmol/L) and time above range (TAR, percentage of glucose values > 10 mmol/L), as well as the minimum and maximum glucose levels, were assessed. To achieve an overview of the diurnal glucose profiles, the glucose average was calculated at each time point for all days of the study period (intervention or baseline), and the area under the glucose curve (AUC_Glu_) was assessed with the trapezoidal rule.

HbA1c was assessed in Labor Berlin (Berlin, Germany), according to standardized procedures.

### 2.6. Statistical Analysis

The study data were collected and managed using REDCap (Research Electronic Data Capture, Version 12.1.1) hosted at Charité—Universitätsmedizin Berlin. SPSS 25 (IBM, Chicago, IL, USA) was used for the statistical analysis. The data were expressed as the mean ± SD when normally distributed and the median (IQR) when not normally distributed. For the analysis of the data distribution, the Shapiro–Wilk test was used. The normally distributed data served for the parametric unpaired or paired Student’s *t*-test, whereas non-parametric Mann–Whitney or Wilcoxon tests were used for the analysis of the not normally distributed values. For the multiple group comparisons of the intervention-induced changes, a one-way ANOVA with Sidak post-hoc test for the normally distributed data and Kruskal–Wallis test with Dunn post-hoc analysis for the not normally distributed data were used. The CGM glucose profiles were compared with a repeated measurement ANOVA (RM-ANOVA). The significance level was set at *p* < 0.05. The visualization of the data was performed using GraphPad Prism software version 5.0 (GraphPad Prism Inc., La Jolla, CA, USA).

## 3. Results

### 3.1. Baseline Characteristics of Study Population

A total of 16 participants consisting of 8 men and 8 women with an average age of 29 (26–34) years, BMI of 26.4 ± 4.3 kg/m^2^, and HbA1c 5.10 ± 0.29% completed this study. No participants with known diabetes were enrolled in the trial. Six participants (three males, three females) were allocated to the BF group, six were allocated to the TRE group (three males, three females), and four participants (two males, two females) were allocated to the control group (Table 1). No differences between the BF, TRE, and control groups in anthropometric measurements and glycemic parameters, including the MSG and glycemic variability indices at the baseline, were observed (Table 1).

### 3.2. Timely Compliance

**Figure 2:**
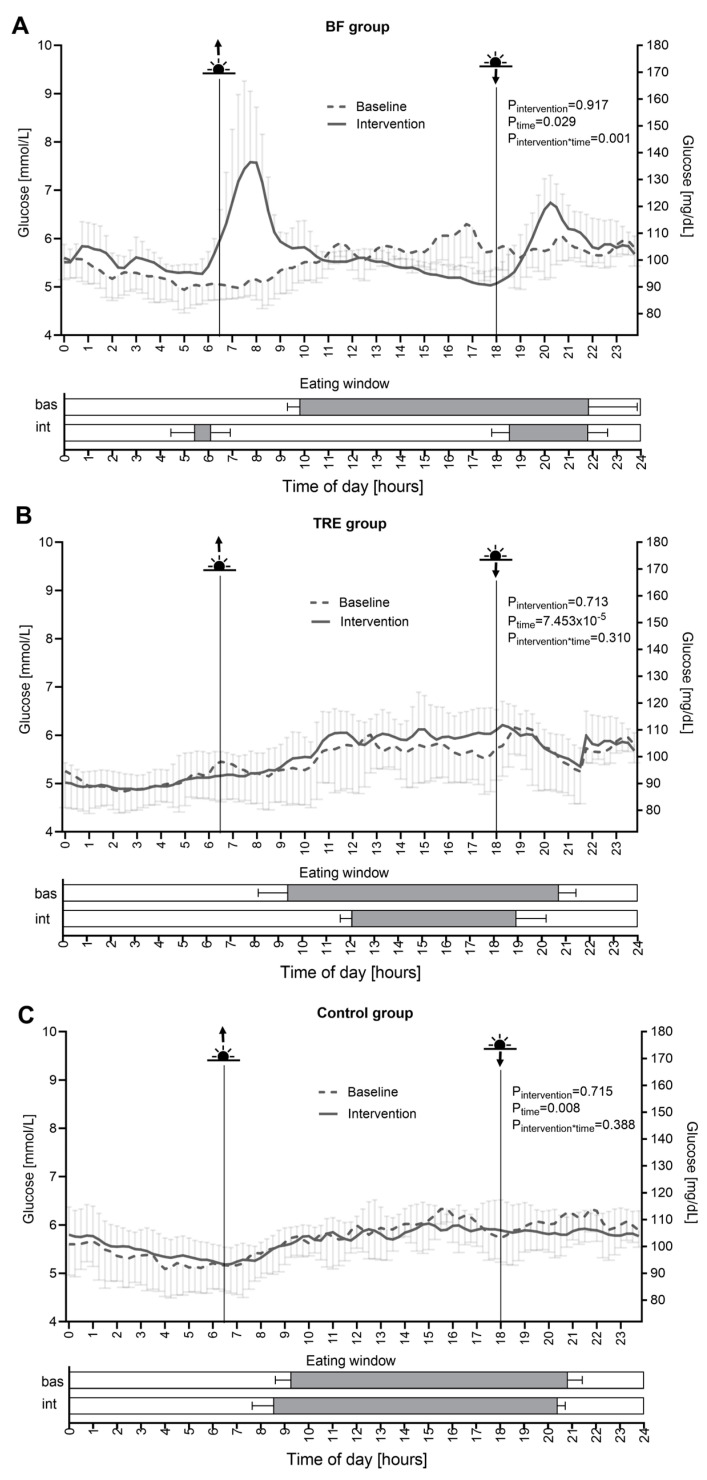
Twenty-four hour glucose profiles and eating windows in the control group (**A**), BF group (**B**), and TRE group (**C**) before and during the intervention. For the glucose profiles (above), the *p*-values show the comparison of diurnal glucose profiles between the baseline and intervention phases, calculated by the RM ANOVA. Eating windows (below) are presented for the baseline (bas) and intervention (int) phases. The data are shown as the mean ± SD.

**Figure 3:**
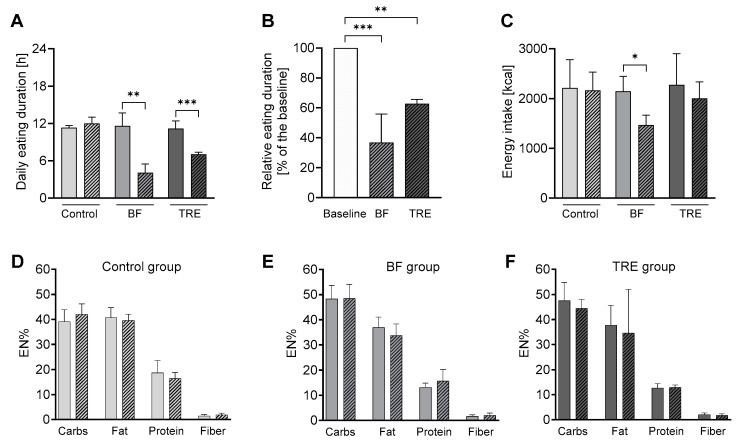
Timely compliance, energy intake, and macronutrient composition in the study groups. (**A**) Daily eating duration; (**B**) reduction in the eating window; (**C**) energy intake; and the energy percent of the macronutrient composition in the control (**D**), BF (**E**), and TRE (**F**) groups. The non-shaded bars depict the values at the baseline; the shaded bars show the values during the intervention. The data are shown as the mean ± SD. * *p* < 0.05; ** *p* < 0.01; and *** *p* < 0.001.

High timely compliance to the prescribed eating timings and durations was achieved, as assessed by adherence to eating before sunrise and after sunset for the BF intervention and by adherence to the 8 h self-selected daily eating window for the TRE intervention. During the BF intervention, the subjects markedly changed their eating regime from eating in the daytime from 9:49 ± 0:32 h till 21:50 ± 2:00 h to eating two times per day from 5:27 ± 01:00 h till 6:07 ± 00:47 h and from 18:32 ± 00:45 h till 21:48 ± 00:48 h, in accordance with the specific sunrise–sunset times (Figure 2A). The total eating window duration was restricted from 11:59 ± 2:09 h to 4:09 ± 1:41 h in the BF group (*p* = 0.008) (Figure 3A). During the TRE intervention, the subjects proceeded to eat in the daytime, only reducing the eating window duration compared to the baseline (Figure 2B). The total eating window duration was restricted from 11:17 ± 1:21 h to 7:03 ± 0:35 h in the TRE group (*p* = 2.94 × 10^−4^) (Figure 3A). Furthermore, the percentage of individual eating time reductions was calculated relative to the baseline. In this context, the eating time window of the baseline was considered to be 100%, as there were no time limitations. The TRE group shortened the relative eating window to 62.8 ± 2.9% and the BF group to 36.9 ± 19.0% compared to the baseline (100%) (Figure 3B). The BF and TRE groups were compliant 100.0 (83.3–100.0)% and 100.0 (91.7–100.0)% of all intervention days, respectively. The control group did not change their habitual eating and fasting timing and duration during the intervention compared to the baseline.

### 3.3. Energy and Macronutrient Intakes

The BF group showed a reduction in energy intake of 677.8 kcal ± 357.6 kcal compared to the baseline (*p* = 0.013), whereas no changes in the energy intake occurred in the TRE and control groups (Figure 3C). The macronutrient and fiber intake, calculated as a percentage of the total energy intake (Figure 3D–F), and the dietary GI (Supplemental Figure S1) were not altered in any group compared to the baseline.

### 3.4. Anthropometric Measurements and Blood Pressure

**Figure 4:**
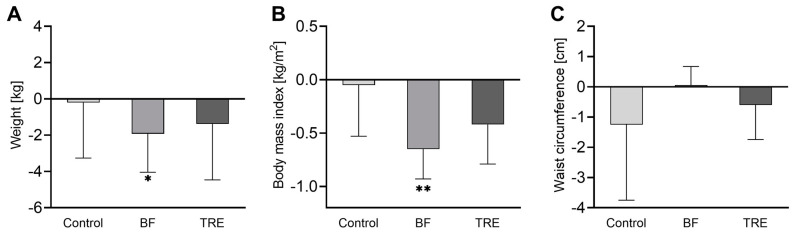
The changes in anthropometric measures in the study groups: (**A**) body weight; (**B**) BMI; and (**C**) waist circumference. The data are shown as the mean ± SD. * *p* < 0.05 and ** *p* < 0.01 in the within-group comparisons of the parameters (after the intervention vs. before the intervention), assessed by the paired *t*-test or Wilcoxon test.

BF induced a decrease in body weight (−1.92 kg ± 0.95 kg, *p* = 0.011) and BMI (−0.65 ± 0.28 kg, *p* = 0.006) (Figure 4A,B), but no effect on waist circumference (Figure 4C). The anthropometric parameters in the TRE and control groups remained unchanged (Figure 4A–C), but no differences were found in the between-group comparison. The systolic and diastolic blood pressure did not change in any group.

### 3.5. Twenty-Four Hour Glucose Profiles and Glycemic Parameters

The BF group showed alterations in the 24 h glucose profiles during the intervention compared to the baseline phase (P_intervention*time_ = 0.001), in contrast to the TRE and control groups, which demonstrated no changes (P_intervention*time_ = 0.310 and P_intervention*time_ = 0.388, respectively) (Figure 2A–C). In agreement with this, in the BF group, the two highest glucose peaks appeared after sunrise and after sunset, whereas the lowest glucose values appeared immediately before sunset (Figure 2A). Correspondingly, the maximum glucose values were markedly increased in the BF group (Supplemental Table S1), whereas they were slightly decreased in the control group during the intervention compared to the baseline phase (1.41 ± 1.04 mmol/L, *p* = 0.039 and −0.55 ± 0.19 mmol/L, *p* = 0.011, respectively). The BF-induced change differed from both of the other groups in the between-group comparison (BF vs. TRE: *p* = 0.022; BF vs. control: *p* = 0.010) (Table 2).

The assessment of the 24 h CGM data shows no changes in the mean sensor glucose (MSG) and area under the glucose curve (AUC_gluc_) in any group (Table 2, Supplemental Table S1). CGM-derived indices of the intra-day (SD, CV, MAGE, and CONGA) and inter-day (MODD) glycemic variability, as well as the LBGI and HBGI remained unchanged in any group compared to the baseline phase and in the between-group comparison. The MAG index decreased in the control group during the intervention (−0.11 ± 0.06 mmol/L/h, *p* = 0.036), but not in the other groups. Furthermore, no changes in the percentages of the TAR and TIR (3.9–10.0 mmol/L) were observed in the CGM data in any group (Table 2, Supplemental Table S1).

## 4. Discussion

This CGM-based study is the first to investigate the impact of religious intermittent dry fasting in the form of BF in comparison to another form of intermittent fasting, TRE, on glycemic control and variability in subjects without diabetes. Surprisingly, despite the reductions in body weight and BMI in the BF group, no alterations in the 24 h mean glucose or the intra- and inter-day glycemic variability indices were found. Similarly, no changes in glycemic control and variability were observed in the TRE group, where the body weight and BMI remained stable after the intervention.

To our knowledge, no long-term CGM-based studies have examined the effect of religious intermittent dry fasting, even in the form of RF, on glycemic control and variability in subjects without diabetes, whereas the metabolic effects of BF have generally been very scarcely investigated so far [5,8]. Till now, only one study conducted short-term CGM for two consequent days before and during RF in seven subjects without diabetes [17]. To close this gap, we conducted a very detailed long-term analysis of glycemic variability using a range of widely used indices of inter- and intra-day variability in subjects without diabetes. Our results showed no alterations in the 24 h mean glucose, AUC_gluc_, intra- and inter-day glycemic variability indices (except for higher maximum glucose values), TAR, and TIR during BF despite the body weight and BMI reductions. These results align with Lesson et al., who also did not find changes in glycemic control in subjects without diabetes using short-term CGM [17]. Taken together, our findings suggest that BF in subjects without diabetes has no clinically relevant negative impact on glycemic control and glycemic variability. This indicates that healthy individuals without diabetes might be metabolically flexible enough to provide good glycemic control even upon BF and the corresponding changes in daily routines and eating and sleeping patterns.

Studies on RF conducted in individuals with well-controlled type 2 diabetes with or without insulin treatment showed similar results. They demonstrated only a transient increase in glucose variability and no changes in glycemic control measures during RF [18,19], although subjects with insulin-treated diabetes showed greater glucose excursions than the other medication groups [17]. Based on these data, Elmalti et al. hypothesized that the initial and temporary increase in glucose variation is related to sudden changes in daily routines and eating and sleeping times, rather than changes in glycemic control or the effectiveness of the diabetes treatment [18]. In contrast, the studies that included only subjects with type 1 diabetes receiving insulin therapy or individuals with both type 1 and type 2 diabetes showed an increased time in hyperglycemia and glucose variability during RF [15,16,17]. Taken together, these data confirm the idea that the risk of severe hypoglycemia upon religious intermittent dry fasting is increased in subjects treated with insulin or with poorly controlled diabetes [2,3,10] but is less or not relevant for subjects without diabetes or with well-controlled type 2 diabetes.

Interestingly, no changes in glycemic control and variability were observed in the TRE group, where the calorie intake was unaltered during the intervention, and the body weight remained stable after the intervention. The data on the TRE impact on glycemic control are controversial. A number of TRE studies showed improvements in glucose metabolism such as lowered fasting glucose levels [30,31,32,33,34], postprandial glucose levels in response to a standard meal or oral glucose tolerance test [35,36], and night-time glucose [37]. HbA1c, as an important indicator of long-term glycemic control, decreased in two trials after a TRE intervention in overweight and obese participants [38,39], while no changes were detected in three other studies [40,41,42]. Few TRE trials that applied CGM showed decreased mean 24 h glucose levels, glycemic excursions [32], and mean fasting glucose levels [35]. The controversial TRE data might be explained by the heterogeneity of the study design (TRE duration and timing of the eating window) and subject cohorts (healthy subjects and subjects with prediabetes or diabetes) [21], as well as different body weight reductions during the intervention. Most TRE trials showed that an improvement in glycemic traits was accompanied by weight loss or did not carefully monitor body weight, which makes it difficult to disentangle the effects of the eating window shortening and weight loss. Our findings indicate that weight loss may be essential for the favorable effects of TRE on glycemic control. The lack of positive changes in glycemic control in the TRE group could be attributed to stable calorie intake and body weight during the intervention. However, further investigation in larger cohorts and different subject categories in carefully controlled trials is needed to distinguish the effects of eating timing and weight loss on glycemic control.

Similar to TRE, the beneficial effect of BF on glycemic control might be expected. The study of Koppold-Liebscher et al. [8] showed that improvements in fasting glucose and HbA1c at the last week of BF were accompanied by weight loss (−2.55 kg) and reductions in the BMI and body fat. In agreement with this, several RF studies demonstrated improvements in HbA1c and fasting or postprandial glucose [11,12,43] in subjects with type 2 diabetes and/or obesity, which, at least in part, might be explained by a reduction in body weight and body fat. A transient improvement in body weight, BMI, and body fat following the RF month has been demonstrated in various original studies and meta-analyses [44,45], with obese subjects exhibiting more weight loss [44], although these findings are also controversial [46]. Nevertheless, in our study, which was accompanied by a minor decrease in body weight (−1.92 kg), no improvements in the CGM-based parameters of glycemic control were found. One possible explanation might be that the study participants without diabetes had good glycemic control before the study started as shown by the low HbA1c levels before the intervention. This leads to the assumption that religious intermittent fasting might have a greater potential for positive changes in subjects with glucose metabolism dysregulation, i.e., prediabetes and diabetes, than in individuals with a normal glucose tolerance. Another explanation might be the shift of the second meal during BF to the late evening, which has a negative effect on metabolic state [47] and might counteract the potential positive effect of weight loss.

The careful documentation of food intake in this present study, which was conducted simultaneously with CGM, allowed an assessment of timely compliance, as well as energy and macronutrient intakes, during the intervention. The quantification of the dietary records confirmed very high adherence to the prescribed eating timing and eating window durations in both the BF and TRE groups, whereas the control group did not change its habitual eating timing (Figure 2). Notably, the eating window for BF was almost as twice as short (4:09 h) as the TRE intervention (7:03 h). The very limited time when food consumption was allowed obviously explains the reduction in calorie intake in the BF group, whereas it remained unchanged in the TRE and control groups. This, in turn, explains the weight loss in the BF but not the TRE and control groups.

The simultaneous analysis of the glucose pattern and dietary intake also allowed for a better interpretation of the observed glucose excursions, as visualized in Figure 2. Specifically, we observed two high glucose peaks after meals consumed both after sunrise and after sunset during the BF intervention. Correspondingly, the maximum glucose values were markedly increased during the BF intervention compared to the baseline, and the BF-induced change differed from those in the TRE and control groups in the between-group comparison. This change in the 24 h glucose pattern during BF was apparently induced by the dramatic shift in eating timing, from daytime food intake to eating two times during the night-time, before sunrise and after sunset. Furthermore, the caloric intake during these two meals was apparently increased compared to the habitual meals during the non-fasting period. This might, at least in part, explain the markedly higher postprandial glucose rise in the BF intervention compared to the daytime meals consumed at the baseline. Notably, while the meals consumed during RF are usually characterized by the consumption of fatty and sugary foods, the BF group in this present study did not show any alterations in the macronutrient composition compared to the baseline, which means that the BF subjects consumed their habitual food during the intervention. Other factors affecting the 24 h glucose pattern during the BF intervention might include changes in the daily routine and sleeping patterns, as well as the water balance and hormonal patterns, described in detail for RF in a recently published review [7]. In particular, diurnal pattern changes of melatonin, cortisol, insulin, leptin, ghrelin, GLP-1, and FGF21 might affect the glucose pattern and glycemic variability in BF. During the TRE intervention, the subjects proceeded to consume food only during the daytime, reducing the daily eating window duration compared to the baseline, but the 24 h glucose pattern was not significantly changed compared to the baseline.

In the context of intermittent fasting, the possible role of the circadian clock needs to be discussed. The circadian clock is molecular machinery that generates 24 h endogenous rhythms of physiology and metabolism, adapting them to day–night changes [47]. Light, food intake, and exercise are important time cues (zeitgebers) that can entrain (shift) the circadian clock [47]. Therefore, dramatic changes in eating, sleeping, and physical activity patterns during BF would apparently affect the clock rhythms, which, in turn, might contribute to rhythm changes in physiological functions. Indeed, Koppold-Liebscher et al. showed that BF markedly advanced the circadian phase of fasting individuals by more than 1 h, which is reversed to normal levels 3 weeks after fasting patterns [8]. Similarly, TRE was also shown to affect endogenous clocks in animal and human studies, stabilizing the circadian clock and increasing the amplitude of circadian rhythms due to the modulation of eating patterns [48,49]. Despite this initial evidence, the interaction of fasting regimens and the circadian clock remains insufficiently investigated and needs to be studied in the future to provide a basis for the development of chrononutritional approaches [7].

This present study demonstrates several notable strengths compared to the prior research. The main strength is the detailed CGM-based analysis of the effects of the religious intermittent dry fasting on glycemic control in subjects without diabetes, which was not previously investigated. This study, for the first time, evaluates a range of inter- and intra-day variability measures in this category of individuals. Additionally, this study is the first to investigate the impact of BF on glycemic control. Another important strength of this present study is the careful control of timely compliance, energy and macronutrient intakes, and 24 h glucose patterns throughout the baseline and intervention phases, providing a comprehensive assessment over approximately 25 days for each subject. We revealed high timely compliance and unchanged macronutrient composition in all groups, and we exactly quantified the energy intake during the intervention. These data were insufficiently controlled in most previously published trials. Finally, a clear strength of this study is that it uniquely compares two distinct forms of intermittent fasting (BF and TRE), while also including a control group.

Our study also has certain limitations. Firstly, the sample size in each group was limited and restricted to subjects without diabetes. According to this, the preliminary data in this article must be considered carefully and need further validation in larger cohorts. Another consideration is that the small sample size limited the utility of subgroup analysis (e.g., subjects with and without obesity). Beyond this, carefully controlled interventional studies are required to compare the glycemic profiles of subjects eating provided standard meals or receiving a balanced diet plan or, at least, to examine a homogenous cohort that has a very similar nutritional composition. Furthermore, monitoring physical activity levels during the study period was not feasible, which prevents a more detailed exploration of the study results. Finally, a follow-up examination after the end of the BF and TRE interventions, which might show some effect on HbA1c at a later stage, was not a part of our study and should be included in future trials.

## 5. Conclusions

Intermittent fasting regimes, such as BF and TRE, do not show a negative (or positive) impact on glycemic control and glycemic variability in subjects without diabetes after a 19-day intervention. The results of this preliminary study need to be confirmed in larger carefully controlled cohorts including metabolically healthy subjects, as well as individuals with prediabetes and type 2 diabetes.

## Figures and Tables

**Table 1:** Baseline characteristics of study population.

	Control Group	BF Group	TRE Group
**Clinical characteristics**			
N	4	6	6
Male	2	3	3
Age [years]	29 (28–35)	31 (22–43)	28 (25–32)
Weight [kg]	85.9 ± 24.2	80.1 ± 18.1	79.5 ± 14.0
BMI [kg/m^2^]	27.4 ± 6.2	26.8 ± 3.9	25.5 ± 3.9
Waist circumference [cm]	89.5 ± 21.0	91.9 ± 17.8	85.2 ± 5.8
SBP [mmHg]	131.0 ± 20.2	132.3 ± 10.5	126.2 ± 12.6
DBP [mmHg]	78.0 ± 13.7	83.8 ± 6.6	85.0 ± 11.1
**Glycemic parameters**			
MSG [mmol/L]	5.74 ± 0.47	5.56 ± 0.30	5.44 ± 0.57
Minimum [mmol/L]	4.96 ± 0.54	4.79 ± 0.35	4.72 ± 0.51
Maximum [mmol/L]	6.77 ± 0.44	6.68 ± 0.38	6.69 ± 0.74
TBR < 3.9 mmol/L [%]	0.20 (0.00–4.39)	0.78 (0.00–2.73)	0.23 (0.00–3.83)
TAR > 10 mmol/L [%]	0.10 (0.00–0.37)	0.00 (0.00–0.81)	0.00 (0.00–0.12)
AUC_gluc_ [min × mmol/L]	8217 ± 673	7964 ± 431	7789 ± 813
SD [mmol/L]	0.81 ± 0.29	0.83 ± 0.10	0.78 ± 0.11
CV [%]	14.2 ± 5.69	14.9 ± 1.55	14.4 ± 1.79
MAGE [mmol/L]	1.03 ± 0.31	1.13 ± 0.05	1.09 ± 0.12
CONGA [mmol/L]	5.23 ± 0.44	4.96 ± 0.27	4.93 ± 0.57
MAG change [mmol/L/h]	1.12 ± 0.26	1.21 ± 0.08	1.09 ± 0.09
MODD [mmol/L]	0.73 ± 0.21	0.75 ± 0.08	0.66 ± 0.06
LBGI	1.28 ± 1.09	1.44 ± 0.61	1.86 ± 1.40
HBGI	0.78 ± 0.58	0.70 ± 0.31	0.59 ± 0.21
HbA1c [%]	5.10 ± 0.29	5.13 ± 0.42	5.07 ± 0.16

Data are shown as mean ± SD when normally distributed and median (IQR) when not normally distributed. BMI, body mass index; SBP, systolic blood pressure; DBP, diastolic blood pressure; MSG, mean sensor glucose; TAR, time above range; TBR, time below range; AUC_gluc_, area under the glucose curve; SD, standard deviation; CV, coefficient of variation; MAGE, mean amplitude of glucose excursions; CONGA, continuous overall net glycemic action; MAG change, mean absolute glucose change; MODD, mean of daily differences; LBGI, low blood glucose index; HBGI, high blood glucose index; and HbA1c, glycated hemoglobin.

**Table 2:** Changes in glycemic control and glycemic variability after the intervention in comparison to the baseline values.

	Control Group		BF Group		TRE Group		*p*-Value Between-Groups Analysis ^c^		
	Changes ^a^	*p*-Value ^b^	Changes ^a^	*p*-Value ^b^	Changes ^a^	*p*-Value ^b^	BF vs. Control	TRE vs. Control	TRE vs. BF
**Glycemic Parameters**									
MSG [mmol/L]	−0.05 ± 0.24	0.710	0.17 ± 0.26	0.220	0.05 ± 0.27	0.690	0.556	0.921	0.871
Minimum [mmol/L]	0.20 ± 0.46	0.460	0.19 ± 0.27	0.187	0.08 ± 0.25	0.520	1.000	0.941	0.939
Maximum [mmol/L]	−0.55 ± 0.19	**0.011**	1.41 ± 1.04	**0.039**	−0.22 ± 0.75	0.556	**0.010**	0.901	**0.022**
TBR < 3.9 mmol/L [%]	−0.11 (−2.56–0.00)	0.180	−0.78 (−2.45–0.67)	0.273	0.08 (−2.85–0.70)	0.715	1.000	1.000	1.000
TAR > 10 mmol/L [%]	−0.07 (−0.31–0.00)	0.180	0.38 (0.00–2.52)	0.109	0.00 (0.00–0.15)	0.317	0.012	0.180	0.209
AUC_gluc_ [min × mmol/L]	−71 ± 343	0.705	241 ± 367	0.216	79 ± 388	0.673	0.548	0.912	0.876
**Glycemic Variability**									
SD [mmol/L]	−0.07 ± 0.14	0.423	0.13 ± 0.18	0.184	−0.02 ± 0.05	0.422	0.155	0.950	0.287
CV [%]	−1.19 ± 3.16	0.507	1.71 ± 2.57	0.226	−0.54 ± 1.34	0.422	0.270	0.971	0.422
MAGE [mmol/L]	−0.14 ± 0.13	0.121	0.19 ± 0.34	0.251	−0.12 ± 0.13	0.113	0.130	0.999	0.133
CONGA [mmol/L]	−0.02 ± 0.22	0.893	0.18 ± 0.26	0.188	0.14 ± 0.31	0.370	0.648	0.792	0.992
MAG change [mmol/L/h]	−0.11 ± 0.06	**0.036**	−0.10 ± 0.16	0.238	−0.16 ± 0.19	0.135	1.000	0.952	0.925
MODD [mmol/L]	−0.04 ± 0.09	0.473	−0.12 ± 0.12	0.098	−0.07 ± 0.07	0.098	0.599	0.957	0.848
LBGI	−0.13 ± 0.72	0.738	−0.28 ± 0.46	0.244	−0.27 ± 0.86	0.523	0.986	0.989	1.000
HBGI	−0.31 ± 0.40	0.220	0.60 ± 0.79	0.164	−0.13 ± 0.15	0.118	0.077	0.953	0.141

^a^ Data are shown as mean ± SD when normally distributed and median (IQR) when not normally distributed. ^b^ Intra-group comparison; *p* < 0.05 by Student’s *t*-test for normally distributed data or Wilcoxon test for not normally distributed data. ^c^ Inter-group comparison by ANOVA test with Sidak post-hoc for normally distributed data and Kruskal–Wallis test with Dunn post-hoc for not normally distributed data. MSG, mean sensor glucose; TAR, time above range; TBR, time below range; AUC_gluc_, area under the glucose curve; SD, standard deviation; CV, coefficient of variation; MAGE, mean amplitude of glucose excursions; CONGA, continuous overall net glycemic action; MAG change, mean absolute glucose change; MODD, mean of daily differences; LBGI, low blood glucose index; HBGI, high blood glucose index.

## Data Availability

The raw data supporting the conclusions of this article will be made available by the authors on request due to privacy reasons.

## Supplementary Materials

The following supporting information can be downloaded at https://www.mdpi.com/article/10.3390/nu16162663/s1. Figure S1: Trial flow diagramme; Table S1: Metrics of glycemic control and glycemic variability before and during the intervention.
