# Supplementary material for: Twenty-Four Hour Glucose Profiles and Glycemic Variability during Intermittent Religious Dry Fasting and Time-Restricted Eating in Subjects without Diabetes: A Preliminary Study
Source: Nutrients. 2024 Aug 12;16(16):2663. doi: 10.3390/nu16162663 (PMC11357114; doi:10.3390/nu16162663)
Supplement: Supplementary file 1 [file nutrients-16-02663-s001.zip › nutrients-3105838-supplementary.pdf]

Supplemental Table S1. Metrics of glycemic control and glycemic variability before and during the intervention.

	Control group			BF group			TRE group		
	**Baseline**	**Intervention**	**p-value[b]**	**Baseline**	**Intervention**	**p-value[b]**	**Baseline**	**Intervention**	**p-value[b]**
**Glycemic Parameters**									
MSG [mmol/L]	5.74 ± 0.47	5.69 ± 0.40	0.710	5.56 ± 0.30	5.73 ± 0.29	0.220	5.44 ± 0.57	5.49 ± 0.42	0.690
Minimum [mmol/L]	4.96 ± 0.54	5.15 ± 0.34	0.460	4.79 ± 0.35	4.98 ± 0.26	0.187	4.72 ± 0.51	4.80 ± 0.32	0.520
Maximum [mmol/L][f]	6.77 ± 0.44	6.22 ± 0.40	**0.011**	6.68 ± 0.38	8.09 ± 1.36	**0.039**	6.69 ± 0.74	6.47 ± 0.56	0.556
Time <3.9 mmol/l [%]	0.20 (0.00 – 4.39)	0.09 (0.00 – 1.83)	0.180	0.78 (0.00 – 2.73)	0.00 (0.00 – 0.94)	0.273	0.23 (0.00 – 3.83)	0.71 (0.15 – 1.33)	0.715
Time >10 mmol/l [%]	0.10 (0.00 – 0.37)	0.03 (0.00 – 0.06)	0.180	0.00 (0.00 – 0.81)	1.00 (0.00 – 3.02)	0.109	0.00 (0.00 – 0.12)	0.00 (0.00 – 0.27)	0.317
AUC_{gluc} [min x mmol/L]	8217 ± 673	8145 ± 569	0.705	7964 ± 431	8205 ± 415	0.216	7789 ± 813	7868 ± 610	0.673
**Glycemic Variability**									
SD [mmol/L]	0.81 ± 0.29	0.74 ± 0.16	0.423	0.83 ± 0.10	0.96 ± 0.28	0.184	0.78 ± 0.11	0.76 ± 0.09	0.422
CV [%]	14.2 ± 5.7	13.0 ± 2.6	0.507	14.9 ± 1.5	16.6 ± 4.1	0.226	14.4 ± 1.8	13.9 ± 1.4	0.422
MAGE [mmol/L]	1.03 ± 0.31	0.89 ± 0.19	0.121	1.13 ± 0.05	1.32 ± 0.36	0.251	1.09 ± 0.12	0.97 ± 0.14	0.113
CONGA [mmol/L]	5.23 ± 0.44	5.21 ± 0.35	0.893	4.96 ± 0.27	5.15 ± 0.24	0.188	4.93 ± 0.57	5.06 ± 0.39	0.370
MAG change [mmol/L/h]	1.12 ± 0.26	1.01 ± 0.22	**0.036**	1.21 ± 0.08	1.11 ± 0.15	0.238	1.09 ± 0.09	0.93 ± 0.11	0.135

MODD [mmol/L]	0.73 ± 0.21	0.69 ± 0.20	0.473	0.75 ± 0.08	0.63 ± 0.10	0.098	0.66 ± 0.06	0.59 ± 0.08	0.098
LBGI	1.28 ± 1.09	1.15 ± 0.60	0.738	1.44 ± 0.61	1.16 ± 0.35	0.244	1.86 ± 1.40	1.59 ± 0.79	0.523
HBGI	0.78 ± 0.58	0.48 ± 0.30	0.220	0.70 ± 0.31	1.31 ± 1.02	0.164	0.59 ± 0.21	0.46 ± 0.23	0.118

[a] Data is shown as mean ± SD when normally distributed and median (IQR) when not normally distributed.

[b] Intra-group comparison; $p<0.05$ by Student's t-test for normally distributed data or Wilcoxon test for not normally distributed data.

MSG, mean sensor glucose; TAR, time above range; TBR, time below range; AUC_{gluc}, area under the glucose curve; SD, standard deviation; CV, coefficient of variation; MAGE, mean amplitude of glucose excursions; CONGA, continuous overall net glycemic action; MAG change, mean absolute glucose change; MODD, mean of daily differences; LBGI, low blood glucose index; HBGI, high blood glucose index.

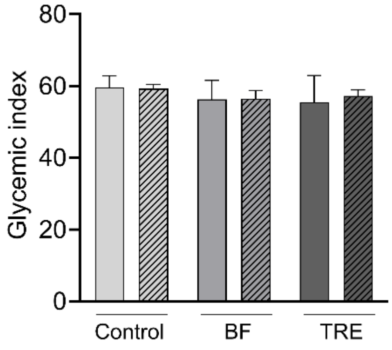

Supplemental Figure S1. Dietary glycemic index (GI) in control, BF, and TRE groups. Non-shaded bars depict values at the baseline, shaded bars show values during the intervention. Data is shown as mean ± SD.
